# Clinical, Morphological and Clonal Progression of VEXAS Syndrome in the Context of Myelodysplasia Treated with Azacytidine

**DOI:** 10.1007/s44228-022-00002-w

**Published:** 2022-05-12

**Authors:** Marco Manzoni, Alessandro Bosi, Sonia Fabris, Marta Lionetti, Simone Salerio, Anna Chiara Migliorini, Francesca Cavallaro, Kordelia Barbullushi, Nicolò Rampi, Vittorio Montefusco, Maria Grazia Alessio, Antonino Neri, Luca Baldini, Mariarita Sciumè, Elena Tagliaferri, Nicola Fracchiolla, Niccolò Bolli

**Affiliations:** 1 Unità di Medicina II, ASST Santi Paolo e Carlo, Dipartimento di Scienze della Salute, Università degli Studi di Milano, Milan, Italy; 2 Hematology Division, Foundation IRCCS Ca’ Granda Ospedale Maggiore Policlinico, 20122 Milan, Italy; 3 Department of Oncology and Hemato-Oncology, University of Milan, 20122 Milan, Italy; 4 Department of Onco-Hematology, ASST Santi Paolo e Carlo, Milan, Italy; 5 Dipartimento di Analisi Cliniche, ASST Santi Paolo e Carlo, Milan, Italy

**Keywords:** VEXAS, Myelodysplastic syndrome, Cytopenia, Inflammation, Oncogenesis

Anemia is the most common cytopenia found in older patients, reaching a prevalence of nearly 30% in patients over 80 years old [1], and has been associated with an increased mortality [2, 3].

A wide range of mechanisms can lead to anemia, ranging from chronic diseases to immunological causes, endocrinopathies and immune deficiencies. Myelodysplastic syndromes (MDS) are also more frequent in the elderly. Differential diagnosis can be challenging for the clinician. While the diagnosis of MDS is based on morphology of the bone marrow (BM), molecular analysis can help through the identification of clonal lesions, classically through karyotyping and Fluorescent In Situ Hybridization (FISH) and, more recently, with the use of Next Generation Sequencing (NGS) [4–6].

Increased use of NGS has allowed the identification in healthy individuals of gene mutations recurrently found in MDS or other myeloid disorders, leading to the definition of a condition termed Clonal Hematopoiesis of Indeterminate Potential (CHIP) [7]. If associated with cytopenias without evidence of dysplasia, the condition is defined as Clonal Cytopenia of Undetermined Significance (CCUS) [8]. To add another layer of complexity, a clonal, non-neoplastic syndrome linked to somatic mutations of the *UBA1* gene in the myeloid compartment was recently described. This syndrome is called VEXAS from the acronym of its main characteristics: vacuoles, E1 enzyme, X-linked, autoinflammatory, somatic. The main clinical features of the VEXAS syndrome are recurrent fevers, relapsing polychondritis, pulmonary and skin involvement, macrocytic anemia, and bone marrow vacuolization restricted to myeloid and erythroid precursor cells [9]. A significant overlap exists between the diagnosis of VEXAS and that of MDS, but which condition arises first and what the interrelationships between the two conditions are is not entirely clear.

In light of these findings, the line between non neoplastic, pre-neoplastic, and overt neoplastic clonal conditions is becoming ever more blurred, and increased awareness is needed by the physician in ordering and interpreting molecular tests. Here, we present an emblematic case of a patient who met the 2016 World Health Organization (WHO) [10] diagnostic criteria for MDS and was successfully treated as such for years. Years into the course of his disease he developed polychondritis and was later found to have VEXAS syndrome and eventually succumbed to disease complications.

The patient was a 77 year-old caucasian male with a history of MDS with multilineage dysplasia since February 2015, classified as very-low risk according to the Revised International Prognostic Staging System (White Blood Cells (WBC) at diagnosis 3.88 × 10^9^/L, absolute neutrophil count 1.65 × 10^9^/L, Hb 129 g/L, platelets 126 × 10^9^/L, normal 46 XY karyotype, bone marrow blasts at diagnosis 1.8%). He was kept without treatment until 2017 when, due to worsening cytopenias (Hb 85 g/L, platelets 62 × 10^9^/L), he was prescribed azacytidine. At the start of treatment, his monthly transfusion need was of 2.5 units of packed red blood-cells. Four months later, the patient reached transfusion independency, which lasted for 14 months. In October 2017, while on azacytidine treatment, he started suffering from recurrent polychondritis involving nose and ears, treated with steroid therapy, and associated with uveitis, erythema nodosum and anti-nuclear antibodies (ANA) positivity (titer 1:160, homogeneous pattern). Red blood-cells transfusion dependency was reinstated on January 2019, initially consisting of 1 packed red blood-cells unit per month, then progressing to 4–7 units per month. In 2020, NGS of a targeted panel of myeloid genes in peripheral blood cells revealed only a mutation in DNA Methyltransferase 3 Alpha (DNMT3A) p.(Arg882Cys), with a variant allele frequency of 48.3%. In January 2021, he also developed platelet transfusion dependency. His clinical course is depicted in Fig. 1. Azacytidine was stopped after 48 cycles, when the patient was admitted to our department with a septic shock. His complete blood cell analysis revealed severe macrocytic anemia (Hb 67 g/L, normal values 135–175 g/L; MCV 97 fL, range 80–94 fL) with severe thrombocytopenia (platelets 18 × 10^9^/L, normal values 130–400 × 10^9^/L) and normal WBC (6.27 × 10^9^/L, normal values 4.80–10.80 × 10^9^/L) with normal formula. C-reactive protein was raised (7.9 mg/dL, cut-off 0.5 mg/dL) with negative procalcitonin, normal renal function and high ferritin levels (30,448 μg/L, normal values 30–400 μg/L) with slightly increased interleukin-6 level (18.7 ng/L–normal values < 10 ng/L). Triglycerides and coagulation factors were normal. Polymerase chain reaction for Epstein-Barr virus (EBV) DNA in peripheral blood revealed an elevated number of copies (41,486 copies/mL, normal values < 250 copies/mL). The patient was initially treated with intravenous fluids, vasoactive amines and broad-spectrum antibiotics. At that point, his hematological history was re-assessed in light of the newly described VEXAS syndrome.

**Fig. 1:**
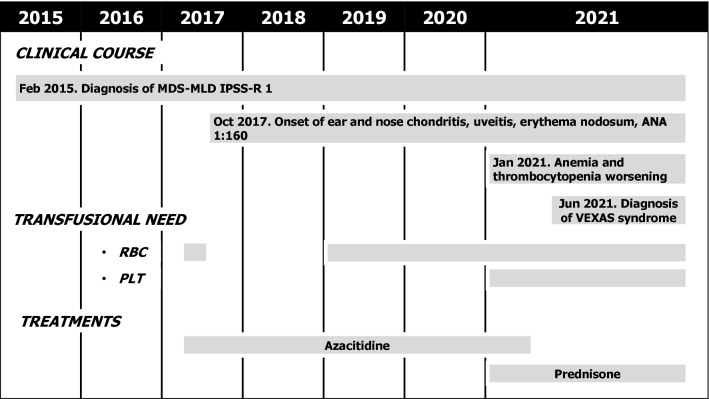
Timeline

It was found that the bone marrow at diagnosis already carried vacuoles in 1.5 and 1.8% of erythroid and myeloid cells respectively (Fig. 2A, C), a proportion which increased to 4.8 and 9.8% at the last assessment (Fig. 2B, C). Furthermore, at the latter point, his blast percentage also increased, and he was found to have increased number of plasma cells, some of which bi-nucleated. The DNMT3A mutation was re-assessed at both time points and was found to be present at a clonal level throughout (Fig. 2D). *UBA1* gene mutation analysis showed a subclonal p.Met41Thr mutation (NM_003334.4:C.122 T > C) at diagnosis, whose allelic burden was possibly increased at the last time-point (Fig. 2D).

**Fig. 2:**
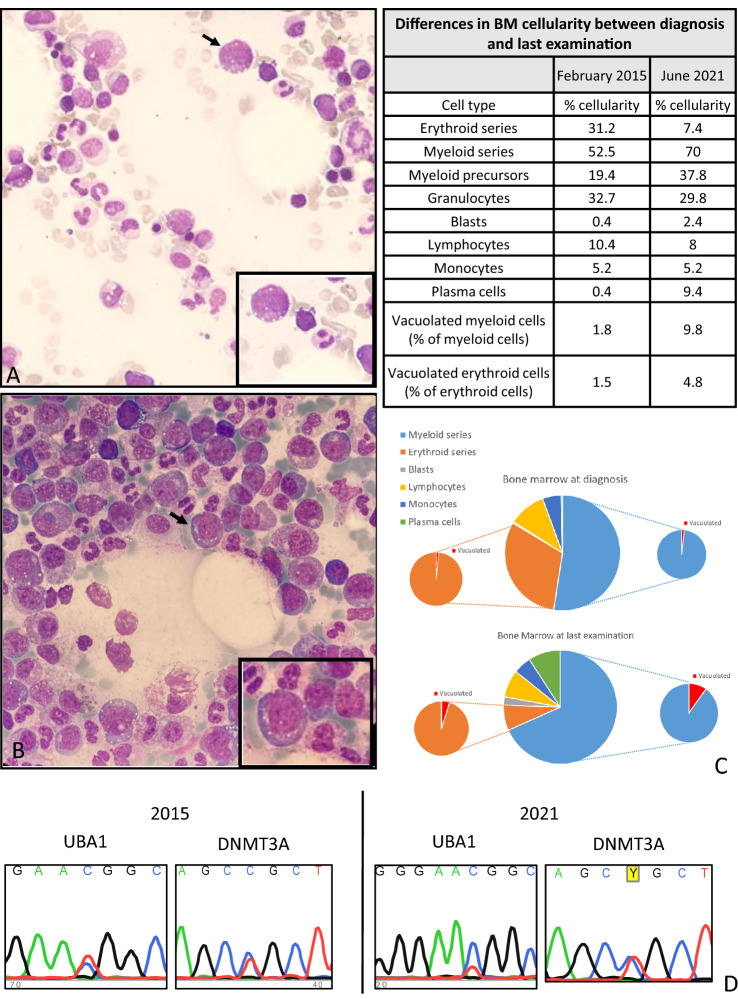
Laboratory, morphological and molecular features

During the subsequent course, the patient showed further infectious complications ranging from a mucosal Herpes simplex virus-1 (HSV-1) reactivation, a suspect P. Jirovecii pneumonia with type I respiratory failure, and positivization of Cytomegalovirus (CMV) viral DNA in peripheral blood. However, due to worsening heart function to New York Heart Association class IV, long-standing cytopenias, and recurrent infections the patient was started on a palliative care pathway and died in hospice 6 years after his initial MDS diagnosis.

Mutations with known oncogenic potential are now known to occur even in healthy individuals, widening the gap between a biologically relevant clonal expansion of mutated cells and a clinically relevant neoplastic condition [11]. In the case of VEXAS, the presence of a clonal, somatic gene mutation not causative of cancer but with large overlap with MDS and *DNMT3A* mutations make the interpretation of molecular tests even more difficult for the clinician. One current hypothesis is that the inflammatory background promoted by VEXAS may favor the emergence of a mutated clone and, thus, the development of MDS, or promote MDS itself [12]. In turn, DNMT3A mutations may favor expansion of a VEXAS clone and even trigger further inflammation [13]. In our patient the *DNMT3A* mutation was clonal throughout the disease course, while the *UBA1* mutation appeared subclonal, and later increased its prevalence, in parallel with a shift in disease phenotype: polychondritis, increased in blast percentage and even plasma cells. This would suggest that the *UBA1* mutation arose in a permissive DNMT3A-mutated genetic background, and its development may have changed the natural history of the disease. Notably, azacytidine was effective in treating the MDS, as expected, given the presence of a *DNMT3A*^*R882H*^ mutation, but had no effect on the autoimmune manifestations of the disease. Furthermore, *UBA1* has a role in suppression of HSV-1 replication [14]. Our patient suffered of multiple reactivations of herpetic viruses (EBV, HSV-1, CMV) which, to date, has not been described in the context of VEXAS syndrome. As additional cases like this are described, more light will be shed on this manifestation as well.

Presumably, *UBA1* will soon be incorporated into NGS-based gene panels for the diagnosis of myeloid malignancies [15], being this part of routine clinical practice in most centers. This will help clarify the pathogenesis of this disease. However, it will also pose additional challenges to the clinician. The opportunity that precision medicine provides is counterbalanced, on the other hand, by an increased risk of over-diagnosis and over-treatment led by a mutation-driven diagnostic process. This is ever more true in a condition whose clinical management is still unclear, especially in the context of co-existing myeloid malignancies.

## Data Availability

Data sharing not applicable to this article as no datasets were generated or analysed during the current study.
